# Contribution of Direct Heating, Thermal Conduction and Perfusion During Radiofrequency and Microwave Ablation

**DOI:** 10.2174/1874120700701010047

**Published:** 2007-09-19

**Authors:** Wolfgang Schramm, Deshan Yang, Bradford J Wood, Frank Rattay, Dieter Haemmerich

**Affiliations:** 1 Div. of Pediatric Cardiology, Medical University of South Carolina, Charleston, SC, USA; 2 Div. Analysis and Scientific Computing, Vienna University of Technology, Vienna, Austria; 3 Dept. of Electrical and Computer Engineering, University of Wisconsin, WI, USA; 4 Dept. Diagnostic Radiology, National Cancer Institute, NIH, Bethesda, MD, USA; 5 Dept. of Bioengineering, Clemson University, Clemson, SC, USA

## Abstract

Both radiofrequency (RF) and microwave (MW) ablation devices are clinically used for tumor ablation. Several studies report less dependence on vascular mediated cooling of MW compared to RF ablation. We created computer models of a cooled RF needle electrode, and a dipole MW antenna to determine differences in tissue heat transfer.

We created Finite Element computer models of a RF electrode (Cooled needle, 17 gauge), and a MW antenna (Dipole, 13 gauge). We simulated RF ablation for 12 min with power controlled to keep maximum tissue temperature at 100 ºC, and MW ablation for 6 min with 75 W of power applied. For both models we considered change in electric and thermal tissue properties as well as perfusion depending on tissue temperature. We determined tissue temperature profile at the end of the ablation procedure and calculated effect of perfusion on both RF and MW ablation.

Maximum tissue temperature was 100 ºC for RF ablation, and 177 ºC for MW ablation. Lesion shape was ellipsoid for RF, and tear-drop shaped for MW ablation. MW ablation is less affected by tissue perfusion mainly due to the shorter ablation time and higher tissue temperature, but not due to MW providing deeper heating than RF. Both MW and RF applicators only produce significant direct heating within mm of the applicator, with most of the ablation zone created by thermal conduction.

Both RF and MW applicators only directly heat tissue in close proximity of the applicators. MW ablation allows for higher tissue temperatures than RF since MW propagation is not limited by tissue desiccation and charring. Higher temperatures coupled with lower treatment times result in reduced effects of perfusion on MW ablation.

## INTRODUCTION

Radiofrequency (RFA) and Microwave ablation (MWA) are used to destroy pathologic tissue by inducing tissue necrosis *via* heating of the targeted tissue. While ablation used for different diseases, here we consider tumor ablation, i.e. treatment of cancer. For tumor ablation, RFA is currently the most common thermal ablation therapy used in a clinical setting. Cryoablation, which is another fairly common tumor ablation modality, uses freezing instead of heat to kill tissue [1]. While surgical resection remains the therapy of choice for liver cancer, few patients have tumors suitable for surgical removal. Current studies suggest that RFA increases the patients 5 year survival rate and performs much better than chemotherapy alone [1, 2]. Radiofrequency ablation and Microwave ablation are clinically used for minimally invasive treatment of inoperable tumors of liver, as well as other organs such as lung, kidney and bone [3]. The most prevalent difference between RF and MW ablation are much higher tissue temperatures obtained with MW with typically shorter application times [4]. Highest temperatures of up to ~100ºC for RFA, and up to ~160ºC for MWA are obtained close to the applicator but temperature drops rapidly with distance from the applicator [5, 6]; the high thermal gradients result in considerable thermal conduction. Knowledge of regions where direct heating and thermal conduction are dominant is especially important when regions close to large vasculature are heated, where thermal conduction alone may not be sufficient to create temperatures in the therapeutic range (>50ºC) [7, 8]. In this study we examined the heating of liver tissue and determined the tissue regions where direct heating and where thermal conduction is dominating for the simulated RF and MW ablation devices. Since our models are designed to include common properties of ablation devices currently used in clinical practice, the clinical relevance of our results is likely, but has to be evaluated in further studies. We simulated RF and MW ablation using finite element method computer models, similar to previous studies [9,-12].

## METHODOLOGY

We employed Finite Element Modeling and Finite Element Analysis to create and solve our experimental setup. We used Abaqus 6.5 for solving the RF model, FEMLab for the MW model, and Matlab 7.0 for further analysis of the generated results. The analysis was performed on a PC with 2GB RAM and a 3.2 GHz Intel Pentium 4 CPU. Both models were designed axi-symmetric due to the symmetry of electrode and antenna. Initial tissue temperature was 37ºC, and this temperature was also applied to the model boundaries. For the model including perfusion according to Pennes’ Bioheat Equation we assumed perfusion to stop when tissue coagulation occurs above ~50 ºC.

### RF-Model (RFM)

We simulated a cooled needle electrode currently in use clinically (Cool-Tip, Valleylab, Boulder, CO). While there are other multi-tined electrodes used clinically, the simple geometry of the needle electrode allowed direct comparison to the MW antenna. The diameter of the exposed electrode is 1.5 mm and the length is 3 cm. This electrode uses internal cooling by circulating water which was simulated by applying 25 ºC as boundary condition on the electrode surface. In the clinical system applied power is controlled by tissue impedance with maximum tissue temperatures of ~100 ºC. In the computer model we controlled applied voltage such that maximum tissue temperature was 100 ºC during the 12 minutes ablation, since above 100 ºC tissue vaporization and charring limit further RF energy deposition.

### RFM Tissue Properties

The tissue properties for the RF-Ablation Model (see Table **1**) were chosen according to [9], thus this simulation results are to be interpreted for a hepatic environment.

### Microwave-Model (MWM)

**Fig. (1):**
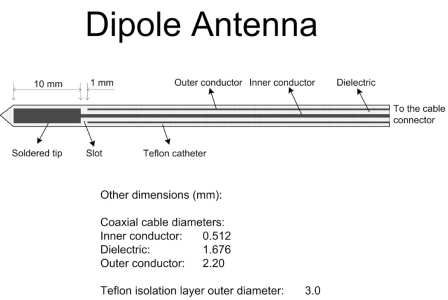
Diagram of the simulated microwave antenna.

We simulated a dipole antenna (2.3 mm diameter, 10 mm dipole length) (Fig. **1**) similar to antennas used clinically [1, 13]. While there are different antenna designs currently used in practice and in research, we chose to employ a dipole antenna due to the fact that this design is well documented and widely used [13]. The antenna was inserted 90 mm into the tissue. 75 W of power was applied for 6 minutes at a frequency of 2.45 GHz. The SAR (specific absorption rate) is significantly affected by changing dielectric tissue properties as tissue water evaporates. Therefore in the electromagnetic (EM) model, tissue water related phenomena, including evaporation, diffusion and condensation, are simulated. The thermal model is based on the expanded Bioheat equation which includes tissue water evaporation at higher temperature. Tissue properties are adjusted depending on changes in water content. This model generates results significantly closer to experimental results than previous static antenna EM models and basic thermal models.

### MWM Tissue Properties

In contrast to the RFM, c (specific heat) and ρ (tissue density) is calculated during the simulation by the following equations [14]:

Density:

(1) $$\rho =v_{w}\times 1000+0.222\times 1300$$

*v_W_* is the tissue water volume per unit volume of tissue, 0 ≤ *v_W_* ≤ 0.778. It is unit less. For normal tissue, *v_W_* = 0.778. Proteins account for 22.2% of the total volume. Density of solid material is 1300 kg/m3 Density of water is 1000 kg/m3.

Specific Heat:

(2) $$c=\sum_{n}w_{n}c_{n}$$

For liver tissue, we assume that tissue is composed of water and proteins. The equation for liver tissue according to water content can be expressed as:

(3) $$C=4200\times w_{w}+1560\times 0.27$$

where *C* is the specific heat [J/g•C], *w_W_* is the remaining tissue water mass per unit mass of tissue, 0 ≤ *w_W_* ≤ 0.73. The equation is based on the assumption that specific heat of solid tissue materials (proteins) is 1560 J/g•C and specific heat of water is 4200 J/kg•C [11, 14, 15].

Bioheat Equation [16]:

(4) $$\rho c\frac{\partial T}{\partial t}=\nabla \cdot k\nabla T+Q_{A}-Q_{p}$$

*ρ* denotes the tissue density.

*c* denotes the specific heat of the tissue.

Energy *Q_A_(W / m^3^)* is applied to the tissue by the applicator (electrode or antenna), resulting in heating of the tissue. Some energy *Q _p_* is carried away by blood perfusion.

(5) $$Q_{p}=\rho_{\mathrm{bl}}c_{\mathrm{bl}}w_{\mathrm{bl}}\left(T-T_{\mathrm{bl}}\right)$$

Where *ρ_bl_[kg / m^3^],c_bl_[J / (kg · K] and T_bl_* are density, specific heat and temperature of the blood, respectively. *T* is the tissue temperature, and *w_bl_* is the blood perfusion *(1/s)*

To evaluate the relation between RFA and MWA we integrated each term over time. Please note, that even though the terms are not independent from each other (e.g. perfusion / conduction) we could determine the relationship between those terms; since we modeled RFA and MWA with and without perfusion; we could directly observe how perfusion and thermal conduction are interrelated.

The temperature increase due to thermal conduction was calculated using the following equation:

(6) $$\Delta T_{\mathrm{cond}}=\int_{t}\frac{\nabla \cdot k\nabla T}{\rho c}\delta t$$

In the same way, we determined temperature increase due to direct heating ( *SAR(W / kg^3^* ) ):

(7) $$\mathrm{SAR}=\frac{Q_{A}}{\rho }$$

(8) $$\Delta T_{\mathrm{SAR}}=\int_{t}\frac{\mathrm{SAR}}{c}\delta t$$

and perfusion:

(9) $$\Delta T_{Q_{p}}=\int_{t}\frac{Q_{p}}{\rho c}\delta t$$

Δ*T_cond_*,Δ*T_SAR_*,Δ*T_Q_p__* were calculated for the 12 minutes RFM simulation , the 6 minutes MWM simulation. We also determined Δ*T_cond_*,Δ*T_SAR_*,Δ*T_Q_p__* for the 0 minutes – 6 minutes and 6 minutes – 12 minutes time spans for the RFM simulation; for the 0 minutes – 3 minutes and 3 minutes – 6 minutes for the MWM simulation. This was done to determine the relative contribution Δ*T_cond_*,Δ*T_SAR_*,Δ*T_Q_p__* in relation to different time spans.

The sum of the three terms in (6) – (8) equates to the total tissue temperature rise:

(9) $$\Delta T=\Delta T_{\mathrm{cond}}+\Delta T_{\mathrm{SAR}}+\Delta T_{\mathrm{perf}}$$

and the final tissue temperature is:

(10) T=37°C+ΔT

### Coagulation Zone Boundary

Even though tissue damage depends both on temperature and time [17], we found in previous studies that the 50 ºC isotherm correlates with coagulation zone boundary within acceptable accuracy [10]. Therefore we used the 50 ºC isotherm to determine coagulation zone boundaries.

## RESULTS

**Fig. (2):**
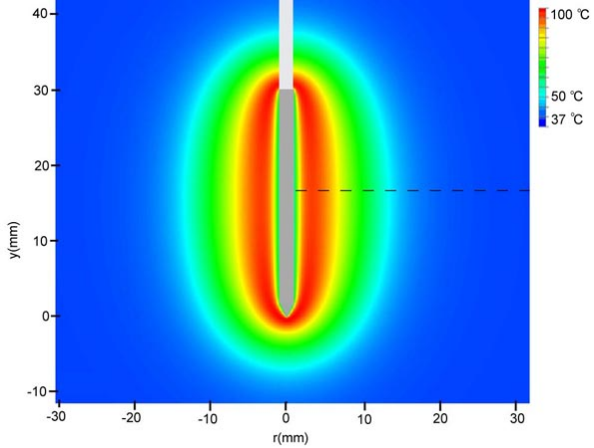
Temperature profile after 12 minutes of RF-Ablation. The dotted line shows the location where analysis was performed for Fig. (**3**). The area with temperatures >50 ^º^C is the ablation zone.

**Fig. (3):**
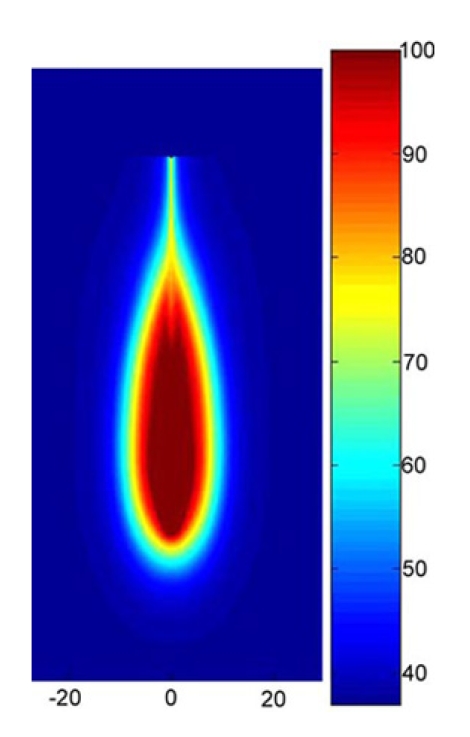
Temperature profile after 6 minutes of MW-Ablation. The dotted line shows the location where analysis was performed for Fig. (**5**). The area with temperatures >50 ^º^C is the ablation zone.

**Fig. (4):**
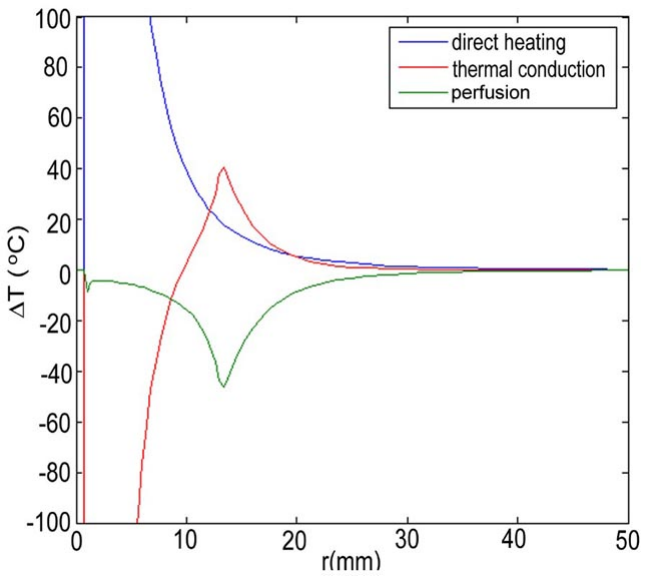
12 minute RF-Ablation with perfusion -Temperature increase ∆T due to direct heating (blue), thermal conduction (red), and perfusion (green). Over the 12 minutes ablation procedure, thermal conduction dominates in the range from 12 to 19 mm radially.

**Fig. (5):**
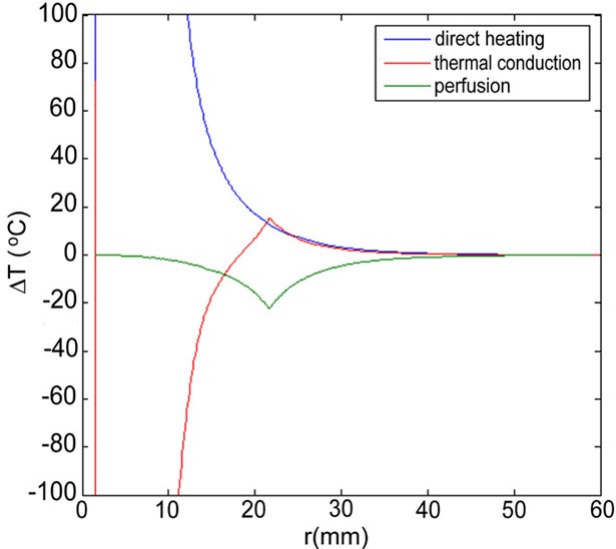
6 minute MW-Ablation with perfusion - Temperature increase ∆T due to direct heating (blue), thermal conduction (red), and perfusion (green). Over the 6 min ablation procedure, direct heating is dominating in the range up to 20 mm radially.

**Fig. (6):**
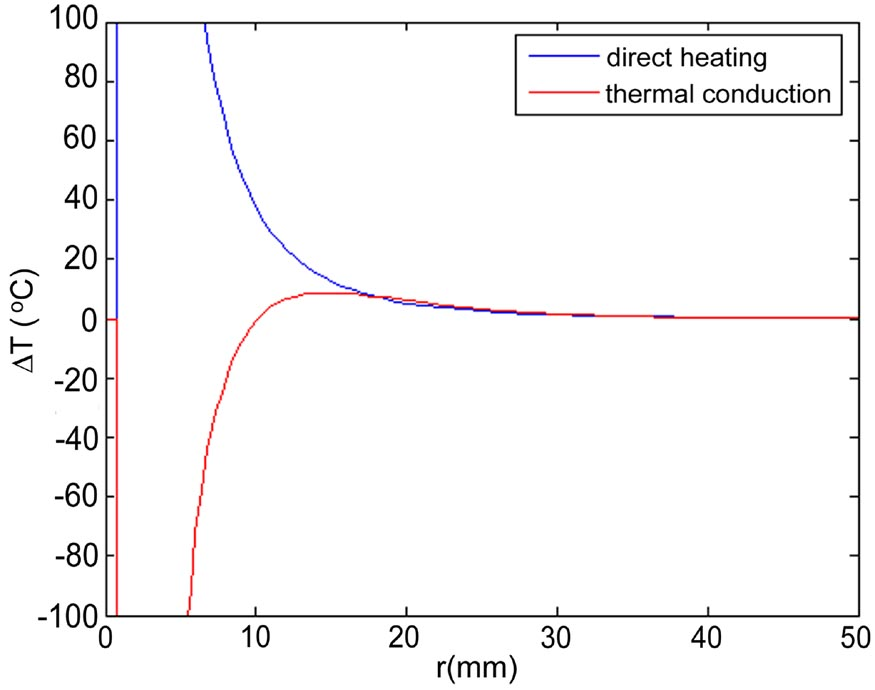
12 minute RF-Ablation without perfusion - Temperature increase ∆T due to direct heating (blue), thermal conduction (red). Due to the absence of a high temperature gradient at the ablation zone boundary because blood perfusion was not included in this model, direct heating is dominating throughout the model.

**Fig. (7):**
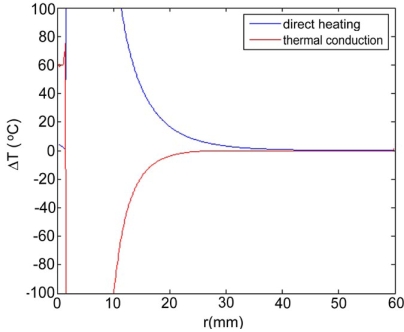
6 minute MW-Ablation without perfusion - Temperature increase ∆T due to direct heating (blue), thermal conduction (red). Due to the absence of a high temperature gradient at the ablation zone boundary because blood perfusion was not included in this model, direct heating is dominating throughout the model.

**Fig. (8):**
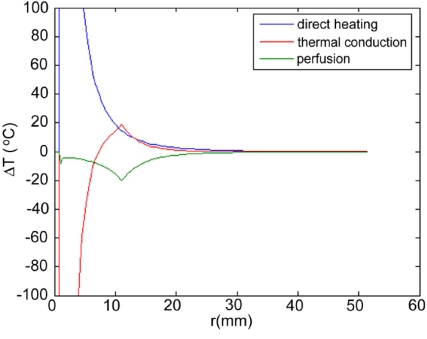
RF Ablation 0-6 Min with perfusion - Temperature increase ∆T due to direct heating (blue), thermal conduction (red), and perfusion (green). Over the first 6 minutes of the ablation procedure, thermal conduction dominates in the range from 9 mm to 11 mm radially.

**Fig. (9):**
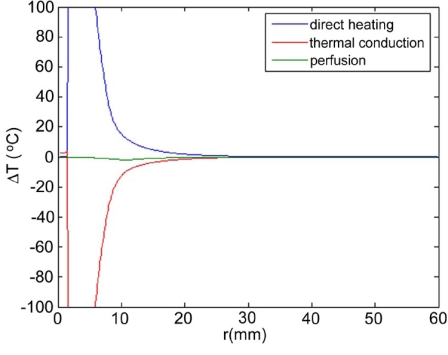
MW Ablation 0-3 Min with perfusion - Temperature increase ∆T due to direct heating (blue), thermal conduction (red), and perfusion (green). Over the first 3 minutes of the ablation procedure, direct heating is dominating everywhere.

**Fig. (10):**
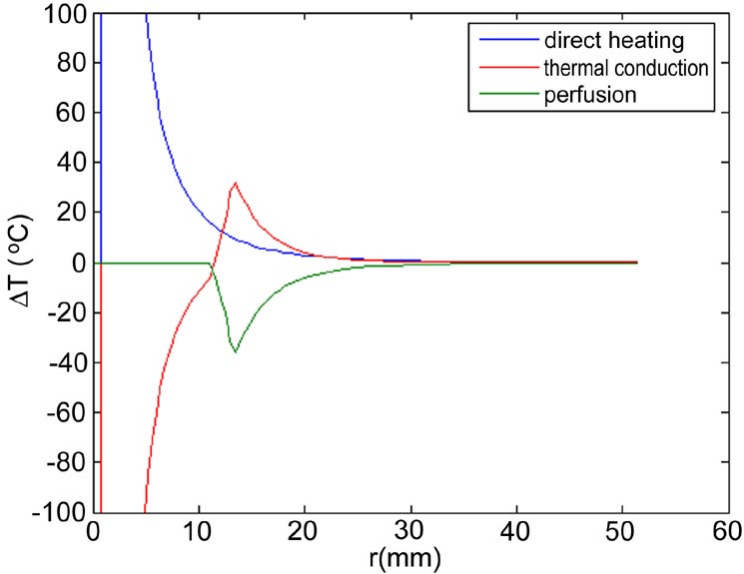
RF Ablation 6-12 Min with perfusion - Temperature increase ∆T due to direct heating (blue), thermal conduction (red), and perfusion (green). Over the last 6 minutes of the ablation procedure, thermal conduction dominates in the range from 12 mm to 20 mm radially.

**Fig. (11):**
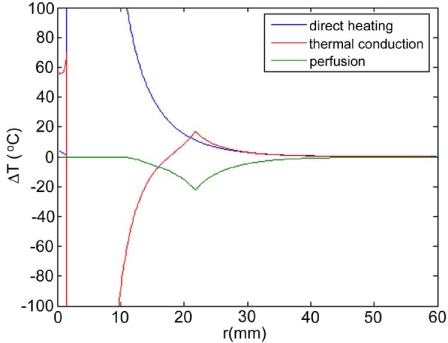
MW Ablation 3-6 Min with perfusion - Temperature increase ∆T due to direct heating (blue), thermal conduction (red), and perfusion (green). In the time between 3 minutes and 6 minutes of the ablation procedure, thermal conduction contributes significantly to tissue heating in the range from 20mm to 30mm radially.

Maximum temperatures were ~100 ºC in the RF model, and 177 ºC in the MW model; tissue charring and tissue vapor does not limit propagation of microwaves, so a higher temperature can be achieved compared to RF. These temperature values are comparable to temperatures measured *in vivo* [5, 6]. The final coagulation zone diameters were 26 mm for the perfused RFM (Fig. **2**), and 36 mm for the unperfused RFM; diameters were 23 mm for the perfused MWM (Fig. **3**) and 26 mm for the unperfused MWM. For the 12 minutes RFM simulation with perfusion, thermal conduction dominates in the range from 12 to 19 mm radially (Fig. **4**). For the 6 minutes MWM simulation with perfusion, thermal conduction is dominating the range >20 mm radially (Fig. **5**). If temperature loss due to blood perfusion is not simulated, direct heating is dominating throughout the RFM (Fig. **6**) and MWM (Fig. **7**). We also determined the time dependent contribution of Δ*T_cond_*,Δ*T_SAR_*,Δ*T_Q_p__*. For the 0 minutes - 6 minutes time span in the RFM (Fig. **8**) direct heating was dominating the whole model, because the influence of blood perfusion has not yet ceased (temperatures <  50 ºC) . For the 0 minutes – 3 minutes MWM (Fig. **9**) time span we can see that thermal conduction dominates the range from 9 mm – 11 mm radially because temperatures > 50 ºC are already reached in this area. For the 6 minutes – 12 minutes time span in the RFM (Fig. **10**) an the 3 minutes – 6 minutes time span in the MWM (Fig. **11**) the relative contribution of direct heating is dominating in the whole model. We also determined the area and the contributed amount of direct heating to the increase of tissue temperature. Fig. (**12**) shows the influence of direct heating in the RF ablation simulation compared to the influence of direct heating in the MW ablation simulation. At each radial location, the fraction of total power within that radius (i.e. integral between 0 and radius) is plotted. For RF, 90% of total power is deposited within 5 mm; for MW, 90% of total power is deposited within 6 mm.

## DISCUSSION

While it has been previously suggested that MWA performs better next to large vasculature due to a larger direct heating area, we found that the volumes of direct heating are similar between RFA and MWA (Figs. **5**, **8**). Our models suggest that the reason MWA is less affected by perfusion is likely that MW heating is not limited by tissue charring around the applicator. This results in higher temperatures during MWA (Fig. **3**). The resulting shorter application times make MWA less susceptible to perfusion effects as demonstrated by our models (Fig. **8**).

Shibata *et al.* [18] performed a study on the effects of invivo microwave and radiofrequency ablation in pig liver. Even though we were not able to compare the ablation zone diameters directly due to the different ablation times used in Shibata *et al*. we did observe the considerably higher ellipticity of the ablation zone shapes note there.

Initially direct heating is dominating everywhere for both RF and MW ablation since tissue temperature is uniform (i.e. no temperature gradient and no heat flux). The integrals of the different heating terms show that for MW ablation, direct heating due to dielectric losses is dominating up to a radius of 20 mm over the 6 minutes MW ablation. Further away thermal conduction and direct heating have similar contributions (Fig. **5**). For RF ablation thermal conduction is dominating in the range from 12 mm to 19 mm radially over the 12 minutes procedure while direct heating due to resistive losses is dominating elsewhere (Fig. **4**). The higher contribution of thermal conduction near the 50ºC-isotherm can be attributed to high temperature gradients occurring at the border of the perfusion zone (50ºC). Tissue cooling due to perfusion is highest just below 50ºC, and zero above 50ºC; this discontinuity in perfusion promotes high thermal gradients, and increased thermal flux near the coagulation zone boundary. Since this discontinuity of thermal gradients is missing in the non-perfused models, the thermal conduction term is significantly smaller near the coagulation zone boundary (Figs. **6**, **7**). Further notable is that thermal conduction contributes little during the first half of the RF and MW procedure (Figs. **8**, **9**), with more significant contribution during the second half (Figs. **10**, **11**). In fact, thermal conduction has similar contribution during both RF and MW ablation for the first 6 minutes (Figs. **5**, **8**).

Therefore, MWA may have clinical advantages close to large vasculature due to the shorter required treatment time compared to RFA.

## CONCLUSIONS

During RF ablation tissue perfusion and thermal conduction contribute more towards tissue cooling and heating compared to MW ablation. This is mainly due to the longer session times necessary with RF ablation, as tissue temperatures are significantly lower compared to MW ablation. The reduced influence of thermal conduction and perfusion due to shorter session times may in part explain why *in vivo* studies, MW coagulation zones are less affected by tissue perfusion compared to RF [6]. The region of direct heating is not significantly different between MW and RF ablation.

## COMPETING INTERESTS

The authors declare that they have no competing interests.

## AUTHORS' CONTRIBUTIONS

WS performed the RF modeling, did the post simulation analysis and wrote part of the paper; DY performed the MW modeling; BJW consulted on clinical issues and wrote part of the paper; FR consulted on mathematical issues and wrote part of the paper; DH planned and supervised the study, and wrote part of the paper.

## Figures and Tables

**Fig. (12):**
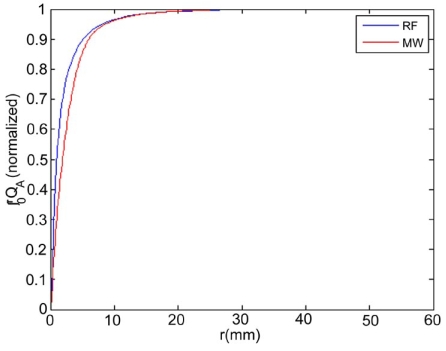
Comparison of the influence of direct heating during RF and MW ablation - Comparison of the influence of direct heating during RF and MW ablation. At each radial distance (r), the fraction of power deposited within that radius is shown. For RF, 90% of total power is deposited within 5 mm; for MW, 90% of total power is deposited within 6 mm.

**Table 1:** Hepatic Tissue Properties for the RF-Ablation Model

*ρ,kgm^-3^*	1060
c,J(kg * K)^-1^	3600
k,W(m* K)^-1^	0.512
*σ,Sm*^-1^ at 500kHz	0.333
